# Kinetics of tick infection by the relapsing fever spirochete *Borrelia hermsii* acquired through artificial membrane feeding chambers

**DOI:** 10.1038/s41598-022-17500-9

**Published:** 2022-08-05

**Authors:** Philip E. Stewart, Sandra J. Raffel, Frank C. Gherardini, Marshall E. Bloom

**Affiliations:** 1 Laboratory of Virology, Rocky Mountain Laboratories, Division of Intramural Research, National Institute of Allergy and Infectious Diseases, National Institutes of Health, Hamilton, MT USA; 2 Laboratory of Bacteriology, Rocky Mountain Laboratories, Division of Intramural Research, National Institute of Allergy and Infectious Diseases, National Institutes of Health, Hamilton, MT USA

**Keywords:** Symbiosis, Entomology

## Abstract

The relapsing fever agent *Borrelia hermsii* is transmitted by the tick *Ornithodoros hermsi*. To study the *B. hermsii*-tick interactions required for pathogen acquisition and transmission we developed an artificial membrane feeding system for *O. hermsi* nymphs and adults that results in a high percentage of engorgement. This system provides the nutritional requirements necessary for the tick to develop, mate, and produce viable eggs. By inoculating the blood with *B. hermsii*, we were able to obtain infected ticks for quantitative studies on pathogen acquisition and persistence. These ticks subsequently transmitted the spirochetes to mice, validating this system for both acquisition and transmission studies. Using this feeding method, a mutant of the antigenic variation locus of *B. hermsii* (Vmp^–^) that is incapable of persisting in mice was acquired by ticks at equivalent densities as the wild-type. Furthermore, Vmp is not required for persistence in the tick, as the mutant and wild-type strains are maintained at similar numbers after ecdysis and subsequent feeding. These results support the theory that Vmp is an adaptation for mammalian infection but unnecessary for survival within the tick. Interestingly, *B. hermsii* numbers severely declined after acquisition, though these ticks still transmitted the infection to mice. This procedure reduces animal use and provides a safe, highly controlled and well-contained alternative method for feeding and maintaining *O. hermsi* colonies. Importantly, this system permits quantitative studies with *B. hermsii* strains through ingestion during the blood meal, and thus more closely recapitulates pathogen acquisition in nature than other artificial systems.

## Introduction

Hindle and Merriman, in their 1912 study “The sensory perceptions of *Argas persicus* (Oken)” observed, “Whilst conducting the experiments on the effect of odorous vapours on both normal ticks and those in which the forelegs had been amputated, we also tried the effect of placing a vessel of hot water beneath the filter paper on which the ticks were walking … many inserted their mouth parts and endeavoured to feed through the filter paper … These results suggested to us a method by means of which ticks might be made to feed on any desired liquid…”^1^. This manuscript presents the first description and use of artificial membrane feeding of ticks that we are aware of. Subsequently, such feeding systems have been developed for both hard ixodid ticks (reviewed by Gonzalez et al.^2^) and soft argasid ticks including *Ornithodoros* species^3–12^. Use of artificial membrane feeding chambers has permitted studies on the nutritional requirements of ticks^13^, the effectiveness of acaracides^14–16^, and the elimination or introduction of microbes to ticks^9,17–21^.

Other methods have successfully introduced bacteria or viruses into ticks, such as immersion, capillary feeding, and injection into the hemocoel or midgut. Although these techniques have their advantages, they also possess some inherent limitations. In the immersion technique, which submerges ticks in a solution of microbes, *Ornithodoros hermsi* infection with *B. hermsii* was relatively low (~ 30%) and the route by which spirochetes enter the arthropod was undetermined^22^. The authors, however, speculated that the microbes were likely taken up through the oral cavity. Capillary feeding is labor- and time-intensive and often yields limited numbers of infected ticks. The microcapillary injection method can produce wounds that may result in an altered physiological state and this approach requires a technical proficiency by the researcher to reduce tick mortality. A significant issue with all three techniques is that they require subsequent feeding on a vertebrate host for the ticks to proceed through the developmental cycle and to assess microbial persistence within and transmission by the vector.

Artificial membrane feeding systems are another alternative that provides numerous advantages. The need for onsite animal usage is alleviated because commercial sources of inexpensive blood are readily available. The method also recreates the natural route of acquisition of many tick-borne pathogens via blood-feeding and entry into the midgut. Additionally, this process offers safe containment of ticks during the feeding process. Specifically, confining the ticks in a feeding chamber avoids the placement of ticks on animals and minimizes the potential of escape, while simultaneously eliminating the possibility of incurring animal bites. Therefore, the use of artificial membrane feeding chambers for vector studies closely mimics the natural progression of pathogen infection and dissemination throughout the tick.

We are interested in pathogen-tick interactions that occur between *Borrelia* species that cause relapsing fever and the soft ticks of the *Ornithodoros* genus. In the U.S., the main agent of relapsing fever is *Borrelia hermsii* (https://www.cdc.gov/relapsing-fever/distribution/index.html) and is transmitted by the tick *Ornithodoros hermsi*. Numerous advances in the genetic manipulation and cultivation have made *B. hermsii* a model organism to study the pathogenesis and ecology of relapsing fever^23–27^.

The relapsing fever *Borrelia* have several fascinating biological characteristics, including a vector specificity that permits only a specific tick species to transmit a cognate *Borrelia* species^28^. This vector specificity resembles that observed for *Leishmania*-sandfly interactions that occurs through recognition of specific lectins in the sandfly midgut by unique lipophosphoglycans produced by the appropriate protozoan species^29,30^. The mechanism for *Borrelia*-*Ornithodoros* specificity remains unknown but relates to the ability of the spirochete species to persistently infect the salivary glands of its cognate vector^31^, in contrast to the midgut interactions that occur between *Leishmania* and the sandfly. Another intriguing characteristic of the relapsing fever *Borrelia* is the antigenic variation system for evading the vertebrate immune system and that permits the reoccurring spirochetemias that have earned the disease its name. When *B. hermsii* is acquired by the tick, the mammalian antigenic system no longer produces a variable major protein (Vmp) and is replaced by the variable tick protein (Vtp)^32^. When the expression locus for the antigenic variation system was genetically disabled producing a mutant strain designated Vmp^–^, the mutant was unable to persist in mice^25^. The regulation of the switching between the Vmp-Vtp loci is not fully understood.

To further our understanding of these and other aspects of *B. hermsii*–*O. hermsi* interactions, we developed an artificial membrane feeding system to provide an alternative method for *O. hermsi* colony maintenance and to introduce the pathogen into the tick. Optimization of the method permitted feeding of adults and later-stage nymphs to repletion, providing all the nutrients necessary for tick development and reproduction. This system was used to introduce both wild-type and the Vmp^–^ mutant strains into *O. hermsi* to further evaluate the contribution of Vmp during the tick infectious cycle.

## Methods

### Bacterial strains and growth conditions

*Borrelia hermsii* WT strain DAH 2E7 passage 3 and the Vmp^*–*^ mutant^25^ were cultured in BSKII medium^33^ at 35 °C under an atmosphere of 5% CO_2_ and 3% O_2_ in a Forma Series II Water Jacket CO_2_ incubator (Thermo Fisher Scientific, Inc., Waltham, MA) with caps loosely attached on the culture tubes.

### Ticks and artificial feeding systems

Ticks were obtained from an *O. hermsi* SIS colony maintained at Rocky Mountain Laboratories that originated from individuals collected from Siskiyou County, CA. Except when being fed, ticks were held in bell jars containing a saturated KCl solution to maintain high humidity levels (~ 85%).

**Figure 1:**
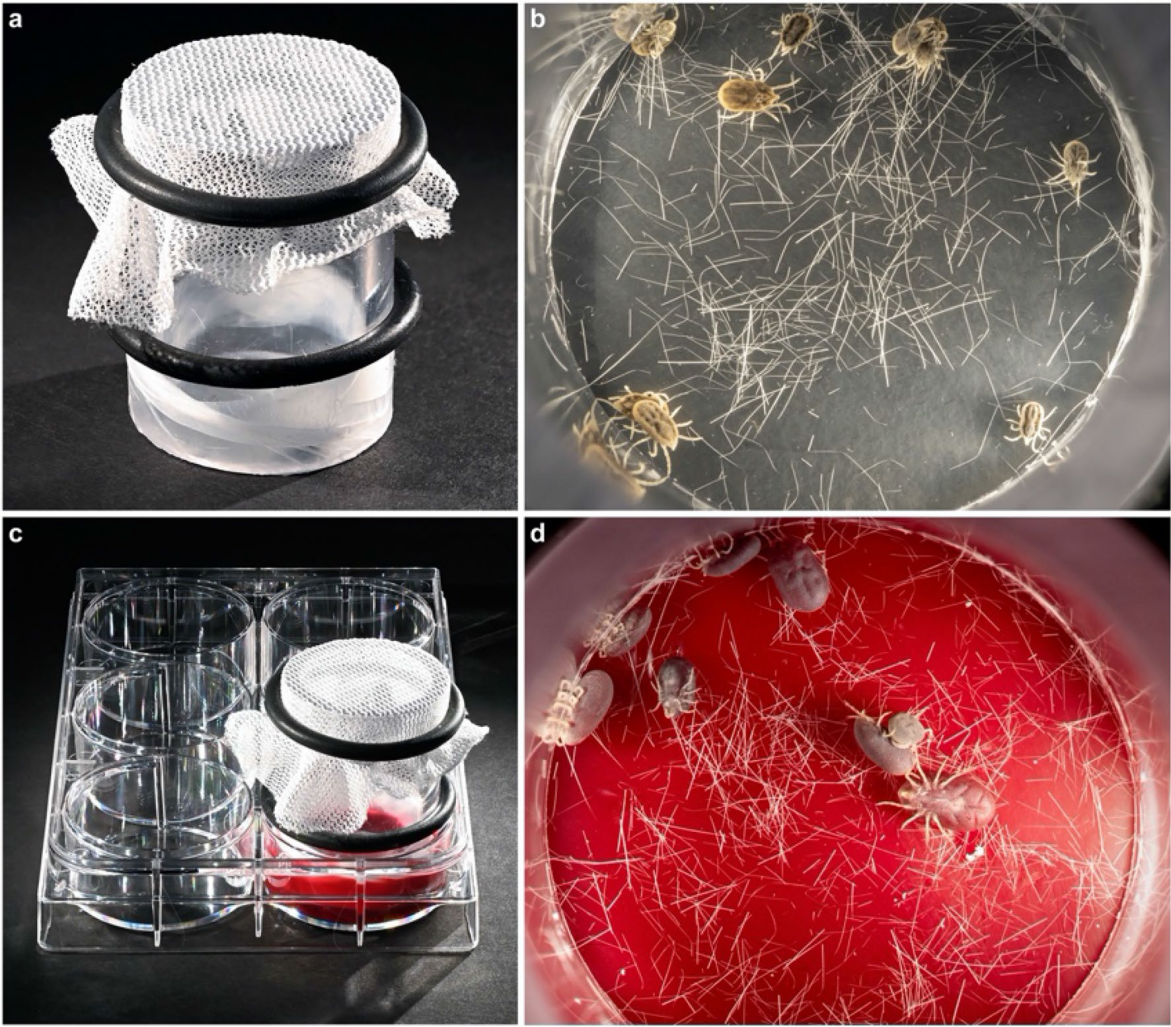
Artificial feeding system for *O. hermsi*. (**a**) The chamber consists of a polycarbonate tube sealed at the bottom with parafilm (that serves as the membrane) and enclosed on the top with mosquito netting. (**b**) Ticks are safely contained within the chamber and the parafilm membrane is lightly covered with mouse hair that may act as a phagostimulant. (**c**) The upper rubber O-ring holds the mosquito netting in place while the lower O-ring can be adjusted to maintain the chamber at the proper height in the blood. (**d**) After incubation, most ticks have fed to repletion.

Various membranes and environmental criteria were tested to obtain conditions for efficient engorgement of *O. hermsi*. The optimized artificial feeding system (Fig. 1) consists of a polycarbonate tube (approximately 2.5 cm inner diameter, and 4.5 cm tall) with Parafilm M stretched tightly across the bottom to act as a feeding membrane and mosquito netting covering the top of the chamber to confine the ticks. The netting was held in place by a tight-fitting rubber O-ring. As a phagostimulant, mouse hair was lightly distributed over the interior surface of the parafilm. Defibrinated bovine or rabbit blood was purchased from HemoStat Laboratories (Dixon, CA, USA) and supplemented with ATP (4 µM) (Sigma-Aldrich, St. Louis, MO, USA) and glucose (2 mg/mL) (Sigma-Aldrich). Blood (4 mL) was aliquoted into each well of a 6-well plate and the chamber, membrane-side down, was set into the blood. A second tight-fitting rubber O-ring on the chamber permitted adjusting the chamber height to position the membrane just below the surface of the blood but above the floor of the plate (Fig. 1c). The feeding system was placed in a dark incubator at 35 °C with 2.5–3.0% CO_2_ for 1–3 h. After feeding, ticks were returned to the humidity-controlled bell jars until needed.

Infecting ticks with *B. hermsii* was accomplished by inoculating the blood reservoir prior to insertion of the chambers. In vitro-cultivated *B. hermsii* were enumerated by darkfield microscopy using a Petroff-Hausser counting chamber (Electron Microscopy Sciences, Hatfield, PA, USA). An actively growing culture of *B. hermsii* was pelleted by centrifugation and resuspended in blood to achieve a final concentration of 1 × 10^6^–6 × 10^7^ spirochetes/mL.

### Quantification of *B. hermsii* in ticks

Immediately after chamber feeding, *B. hermsii* densities in individual ticks and from the blood reservoir were determined by colony enumeration in solid medium, as previously described^26^. Briefly, engorged, infected ticks were surfaced sterilized by washing sequentially in 3% H_2_O_2_, 70% ethanol, and sterile water. Ticks were then pulverized using a sterile pestle in a 1.5 mL Eppendorf tube with 0.1 mL BSKII, then raised to 1 mL final volume. Aliquots of this suspension were added to plating medium^26^ that was allowed to solidify at room temperature prior to incubation at 35 °C in 5% CO_2_ and 3% O_2_ using a Forma Series II Water Jacket CO_2_/O_2_ incubator (Thermo Fisher Scientific, Inc., Waltham, MA, USA). Plates were incubated for 10–14 days after which colony forming units were counted and total spirochete densities were calculated for each tick and the blood reservoir. Statistical analysis was performed using GraphPad Prism software version 9.1.1 for macOS (GraphPad Software, San Diego, CA, USA).

### Tick-mouse transmission studies

Ticks initially infected via the artificial feeding membrane system were held in bell jars containing a saturated solution of potassium chloride. After molting, infected ticks were allowed to feed on female C3SnSmn.Cg-*Prkdc*^*scid*^/J mice (The Jackson Laboratory, Bar Harbor, Maine) to assess the competence of the ticks to transmit *B. hermsii* to a vertebrate host. Mice were anesthetized with pentobarbital (0.5 mg/10 g body weight) (Abbott Laboratories, North Chicago, IL) via intraperitoneal injection. Three *O. hermsi* ticks were fed on each of five SCID mice. Mice were monitored on days 4–7 post-infestation for spirochetemia by examining blood samples by thin-smear darkfield microscopy for the presence of motile *B. hermsii* among the red blood cells. When a spirochetemia was detected, mice were euthanized.

### Ethics statement

All animal experiments were conducted at the Rocky Mountain Laboratories, in accordance with the guidelines of the The Rocky Mountain Laboratories’ Institutional Animal Care and Use Committee (RML ACUC) and with the ARRIVE guidelines (https://arriveguidelines.org). Personnel were trained and certified in accordance with the guidelines of the Association for Assessment and Accreditation of Laboratory Animal Care, International and the Office of Laboratory Animal Welfare. Mice were cohoused in HEPA-filtered cages with nesting material, and commercial food and water were available ad libitum. Humane endpoint criteria were specified in the RML ACUC-approved animal study protocols (ASP 2018-061 and 2021-059) to determine when mice should be humanely euthanized.

## Results

### Optimization of an artificial membrane feeding system for *O. hermsi*

Multiple variables were tested in the development of an efficient feeding chamber for *O. hermsi* ticks, including membrane composition, environmental conditions, and kairomones and phagostimulants (Table 1). In all, 92 of 134 ticks (69%) in 11 trials fed to repletion on the artificial membrane (Table 1). The most efficient conditions for tick engorgement were obtained using parafilm M membranes with the addition of mouse hair, glucose and ATP in the blood, and incubation at 35 °C and 5% CO_2_. Although we did not assess the individual requirement of each component, higher feeding efficiencies were obtained with these additions. ATP is a phagostimulant for inducing tick feeding^34,35^, while hair^6^ and CO_2_ may act as kairomones. Ticks fed through both silicone-based and parafilm M membranes, but the latter is easier and cheaper to use and produced consistently high proportions of engorgement. When used with these conditions, the artificial membrane feeding chambers (Fig. 1) routinely induced 80–92% of the ticks to feed to repletion (Table 1). Usually, 100% of the engorged ticks survived through ecdysis.

**Table 1:** Tick feeding efficiencies and survival results using different artificial membranes and environmental conditions.

Tick stage	Tick #	# FED ticks (%)	% Survived molt	Chamber conditions	Notes
Nymph	13	4 (31)	100	Silicone membrane, bovine blood with 4 µM ATP, 37 °C water bath	
Nymph	13	4 (31)	75	Silicone membrane, rabbit blood with 4 µM ATP, 37 °C water bath	
Nymph	14	6 (43)	100	Parafilm membrane, rabbit blood with 4 µM ATP, 37 °C water bath	
Adult	4	4 (100)	100	Parafilm membrane, rabbit blood with 4 µM ATP, 37 °C water bath	Laid eggs, hatched to larvae
Nymph	10	3 (30)	100	Parafilm membrane, rabbit blood with 4 µM ATP, 37 °C water bath	
Nymph	14	12 (86)	58	Parafilm membrane, bovine blood with 4 µM ATP, + Glucose + mouse hair, 35 °C /2.5–3% CO_2_	
Nymph	10	8 (80)	100	Parafilm membrane, bovine blood with 4 µM ATP, + Glucose + mouse hair, 35 °C/2.5–3% CO_2_	2 smaller nymphs did not feed
Nymphs and adults	26 (from 3 independent feedings)	24 (92)	100	Parafilm membrane, bovine blood with 4 µM ATP, + Glucose + mouse hair, 35 °C/2.5–3% CO_2_; *B. hermsii* WT inoculated into blood	Laid eggs, hatched to larvae
Nymphs and adults	30 (from 3 independent feedings)	27 (90)	100	Parafilm membrane, bovine blood with 4 µM ATP, + Glucose + mouse hair, 35 °C/2.5–3% CO_2_; Vmp^-^ mut inoculated into blood	Laid eggs, hatched to larvae

Nymphs that fed through the artificial membranes molted and developed to the next life stage, while adults that fed in this manner mated and produced viable eggs (Table 1). Therefore, this system provides all the necessary nutritional requirements for ticks to progress through the lifecycle from a nymph to the adult and to reproduce. However, we were unsuccessful at inducing the larvae and first-stage nymphs to feed under any conditions tested (data not shown).

### *O. hermsi* can acquire* B. hermsii* via artificial membrane feeding

**Figure 2:**
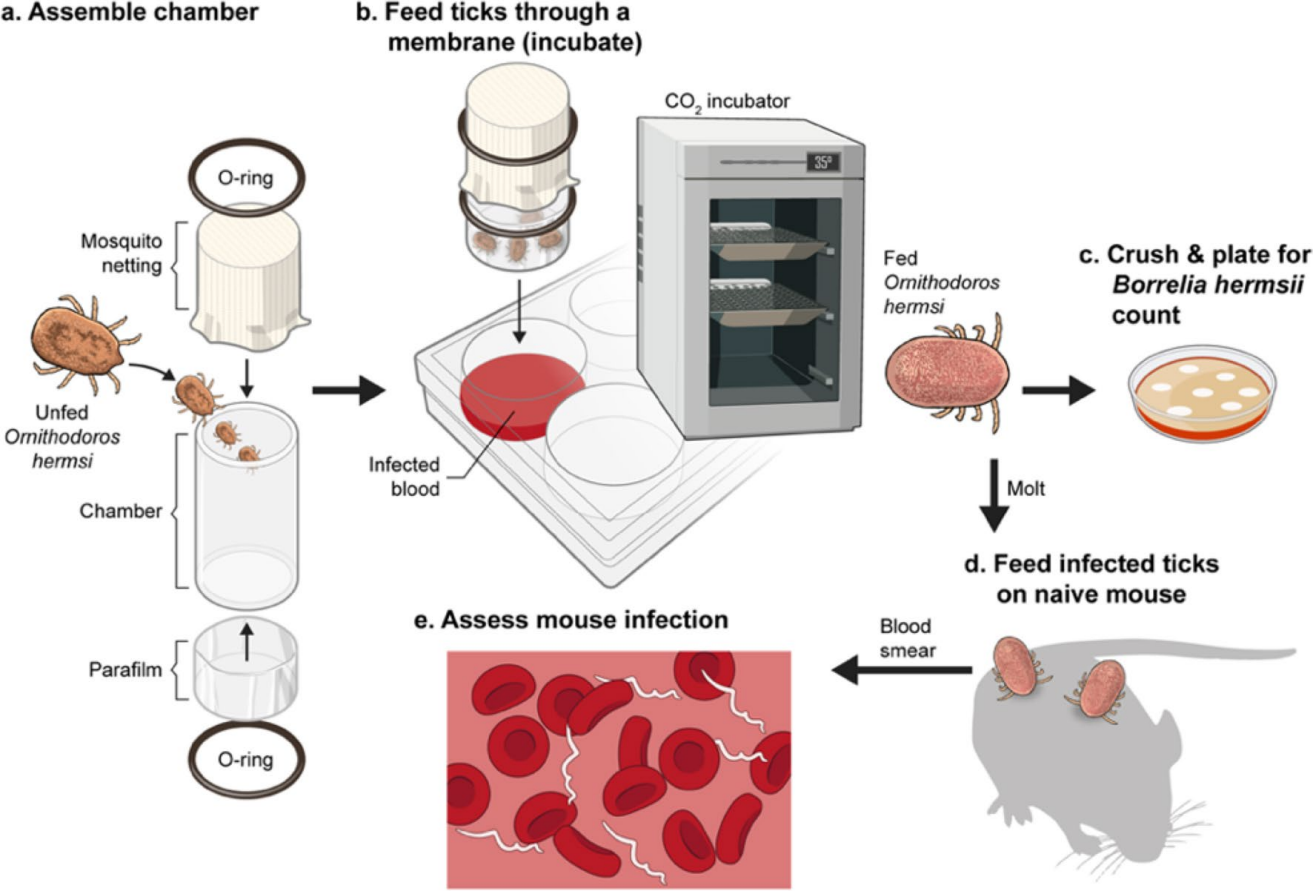
Overview of experimental strategy. (**a**) *O. hermsi* ticks were placed in the artificial membrane chambers and (**b**) allowed to feed on infected blood. (**c**) A portion of the fed ticks were pulverized in liquid medium and diluted into solid medium to calculate spirochete densities. (**d**) The remaining ticks were allowed to molt and then to feed on naïve mice, after which *B. hermsii* transmission was assessed by examining mouse blood for spirochetemias (**e**).

The mouse-tick infection cycle of *B. hermsii* can be accurately modeled with laboratory mouse strains and tick colonies. However, the ability to study tick-specific phenotypes using this lab model is compromised when studying mutants incapable of infecting mice^25,36^. Artificial membrane feeding chambers provide a useful method of producing pathogen-infected ticks via the route of blood-feeding^9,17–19,21^. Figure 2 shows the experimental strategy used in this study. The genetically engineered Vmp^–^ mutant, is unable to undergo antigenic variation and does not cause relapses in mice, making it difficult to obtain infected ticks by feeding on inoculated mice^25^.

**Figure 3:**
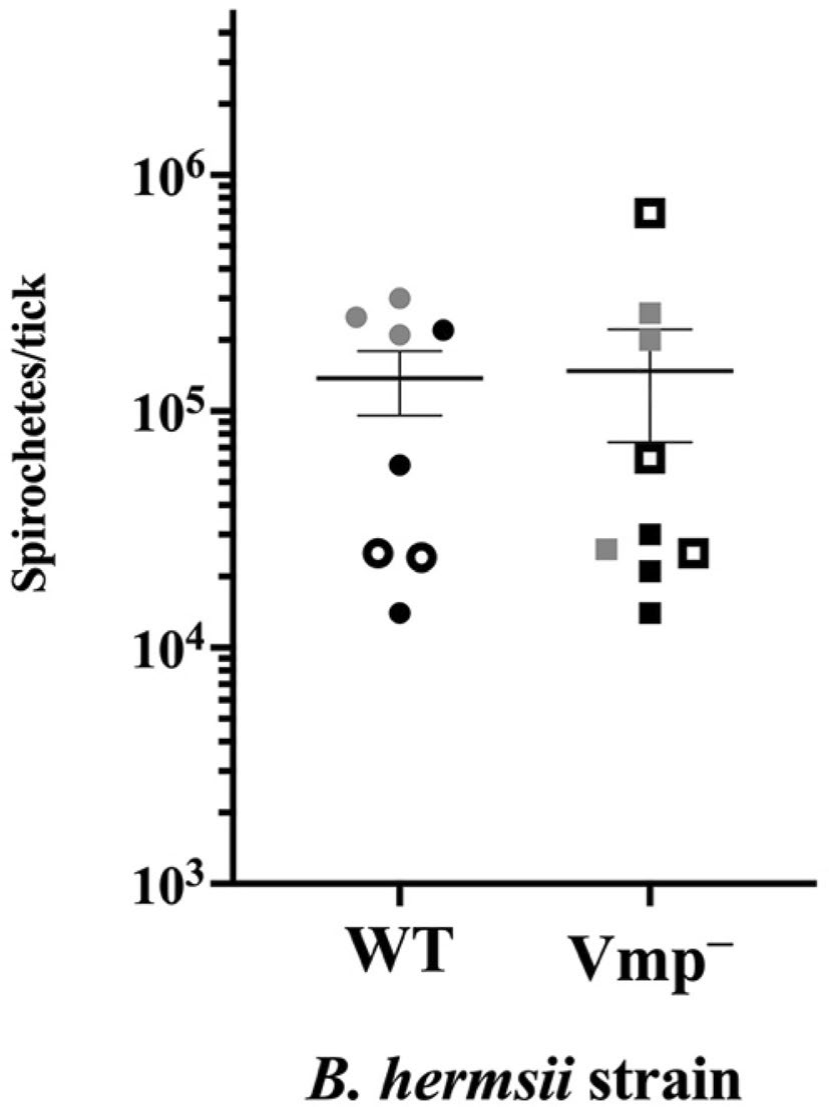
Numbers of *B. hermsii* spirochetes in *O. hermsi* ticks post-feeding. Immediately after feeding through artificial membranes on infected blood, individual ticks were crushed and spirochete densities determined by colony count on solid medium. No significant difference was observed between acquisition of the WT strain and the Vmp^–^ mutant as calculated by an unpaired, 2-tailed T-test. Each symbol represents *B. hermsii* densities within an individual tick, and differences in shading indicates independent feeding experiments (3 total). Bars represent the mean ± the standard error of the mean.

Using the artificial membrane feeding chambers we infected *O. hermsi* ticks with both the WT and Vmp^–^ strains of *B. hermsii*. In vitro cultivated spirochetes were inoculated into the blood reservoir and ticks were allowed to feed to engorgement. After feeding was completed, aliquots from the blood and from 2 to 3 crushed ticks from each chamber were added to plating medium to confirm spirochete concentrations. Spirochete concentrations remaining in the blood varied between 1 × 10^6^ and 6 × 10^7^ *B. hermsii*/mL, depending on the experiment, a concentration range observed in the blood of spirochetemic mice^25,37,38^. These results demonstrated that the Vmp^–^ mutant was acquired by ticks at comparable densities to that of WT (Fig. 3), thus confirming that Vmp does not contribute to acquisition of *B. hermsii* by *O. hermsi* ticks^25^.

### Membrane-fed ticks maintain and transmit *B. hermsii*

After molting to late-stage nymphs or adults, we used *O. hermsi* previously infected by blood-feeding through the artificial membrane chambers to assess their ability to transmit *B. hermsii* to mice. Because the Vmp^–^ mutant does not relapse or reach the densities of WT *B. hermsii* in immunocompetent mice^25^, we used C3SnSmn.Cg-*Prkdc*^*scid*^/J (SCID) mice. The lack of a humoral immune response in SCID mice permits relapsing fever spirochetes to attain persistently high densities, making infection easier to detect by darkfield microscopy for *B. hermsii* in the blood.

**Figure 4:**
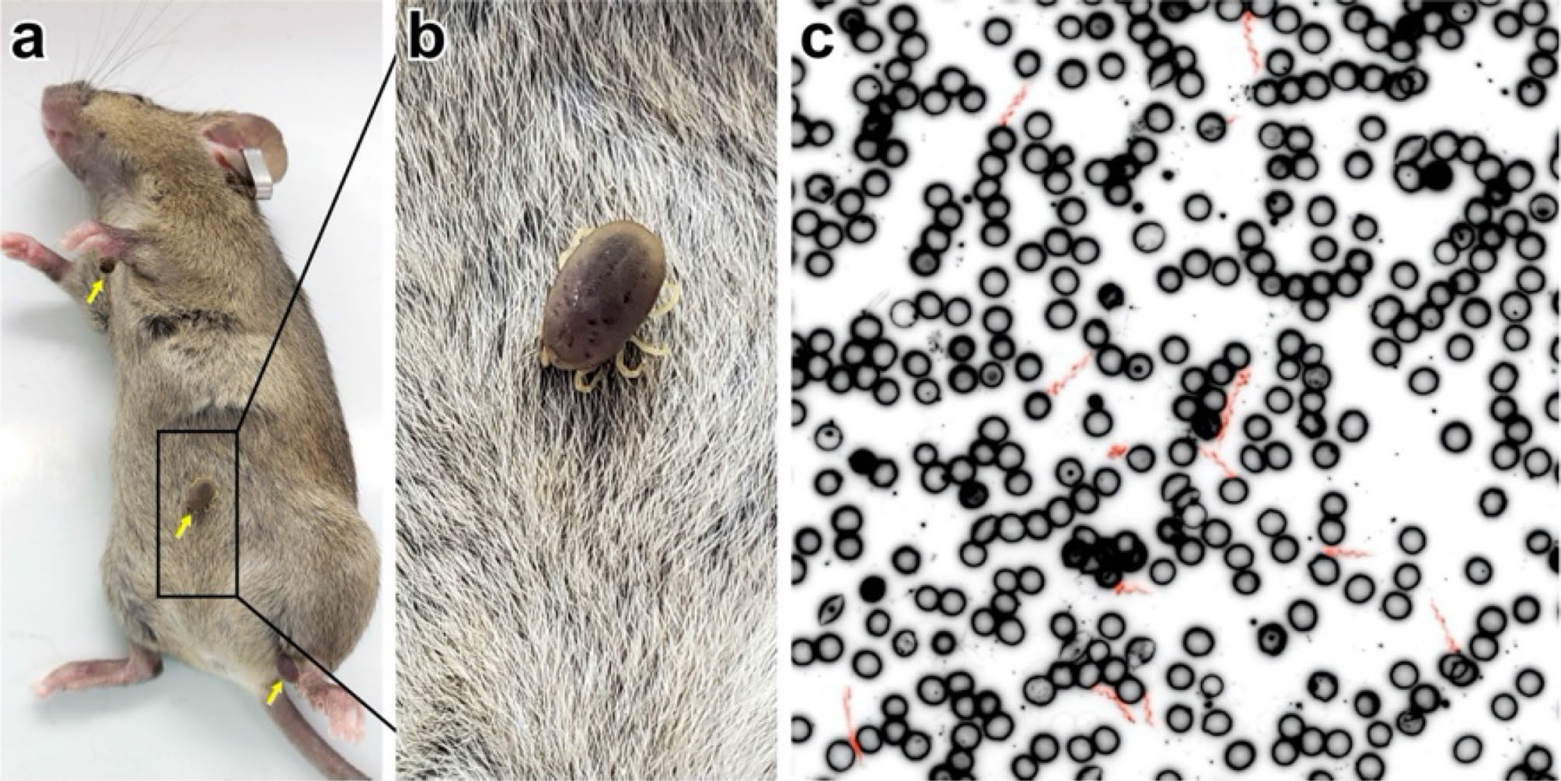
Transmission of *B. hermsii* by tick bite. (**a**) Three infected ticks (yellow arrows) were allowed to feed to repletion on individual SCID mice. The boxed area is enlarged (**b**) to show a feeding *O. hermsi* tick. (**c**) Spirochete transmission to the mice was confirmed by visualizing the spirochetes among the red blood cells by thin smear. To enhance visualization of *B. hermsii* in blood, an inverse image was used and spirochetes were highlighted in red using Adobe Photoshop. Representative images are shown.

In a typical trial, three ticks were placed on individual SCID mice and allowed to feed to repletion; infection was then affirmed by darkfield microscopy for the presence of spirochetes in the blood (Fig. 4). All six mice fed upon by WT-infected ticks became spirochetemic, but only two of four SCID mice total became infected with the Vmp^–^ strain. The two mice that did not become infected were fed upon by one or two ticks only. Hence, consistent infection of SCID mice required 3 ticks per animal, and was less successful when 1–2 infected ticks fed per mouse. Both *B. hermsii* strains were transstadially maintained by *O. hermsi* ticks infected by feeding through artificial membrane chambers and transmitted the infection to SCID mice. Therefore, this model system mimics the natural tick-mouse infectious cycle.

**Figure 5:**
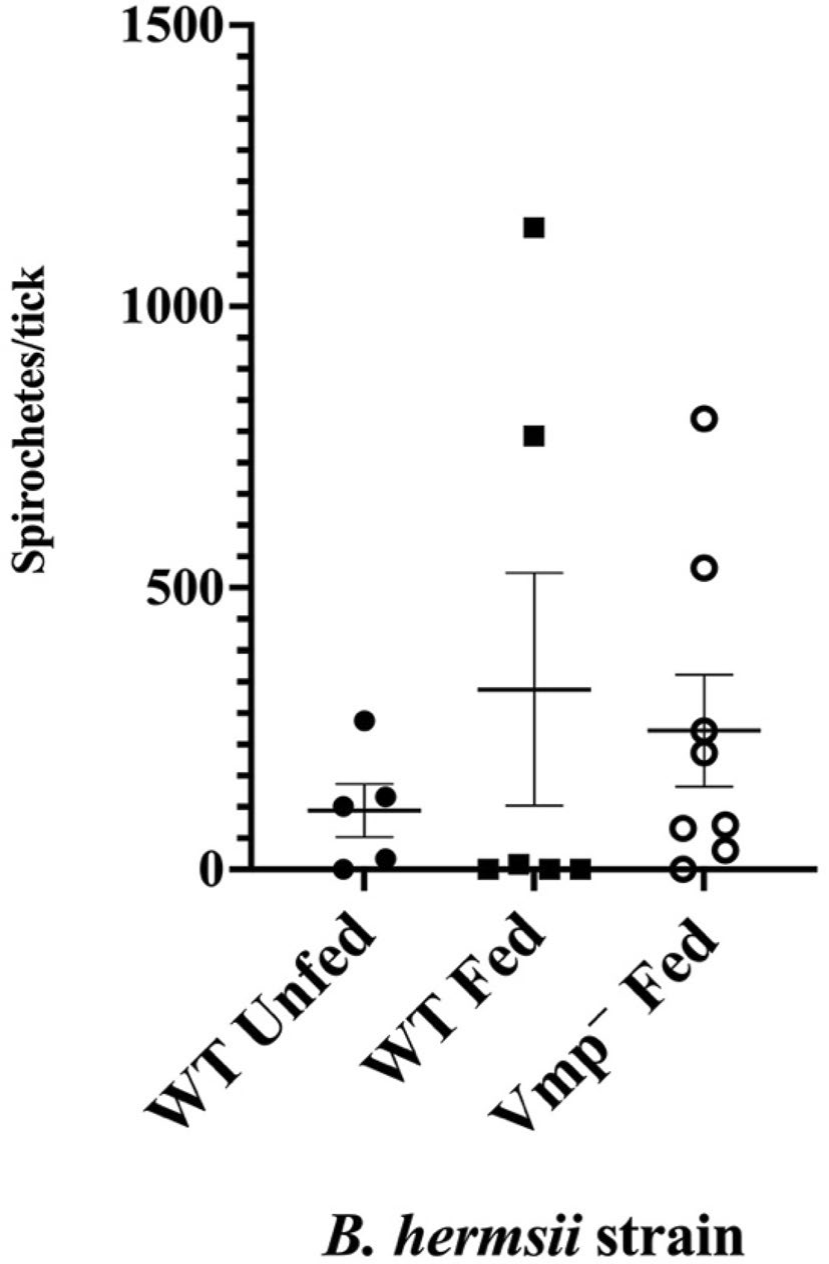
*B. hermsii* densities within the tick decrease following acquisition. Ticks that had acquired the infection by feeding through an artificial membrane were sampled after molting (WT Unfed), or after a subsequent bloodmeal on uninfected SCID mice (WT Fed and Vmp^–^ Fed). Individual ticks were crushed and spirochete densities determined by colony count on solid medium. Each symbol represents *B. hermsii* densities within an individual tick. No significant difference was observed between WT Fed and the other two samples, as calculated by an unpaired, 2-tailed T-test. Results from two independent feeding experiments are shown. Bars represent the mean ± the standard error of the mean.

Interestingly, *B. hermsii* densities within ticks were very low following the molt and subsequent feeding (Fig. 5), whether the ticks were fed or unfed, compared to initial densities of spirochetes detected in ticks after acquisition (Fig. 3). This indicates that the reduction in spirochete numbers was not due to effects from the subsequent bloodmeal but to changes occurring either during digestion of the initial bloodmeal or during the molt. Both the WT and Vmp^–^ mutant strains had similar declines and were not significantly different from each other.

## Discussion

*Ornithodoros* ticks are widespread globally, native to both the old world and the new. Some of these tick species have evolved a unique symbioses with specific members of the relapsing fever clade of *Borrelia*, and new symbiotic species are still being identified^39^. Occurring in Africa, Asia, Europe and the Americas, relapsing fever produces flu-like symptoms including fever, nausea, chills, aches, and fatigue^40^. More severe clinical features include diarrhea, respiratory issues, neurological complications that can result in death, and in utero infections leading to abortions or still births.

The ability of relapsing fever *Borrelia* to evade the host immune system and cause relapses is due to the antigenic variation of the Vmp proteins, although in most cases the host eventually clears the pathogen^41^. A mutant incapable of expressing Vmp is attenuated for mammalian infection and rapidly cleared by the host^25^. Identification of mutants attenuated for host infection (e.g. Vmp^–^) are critical to understanding the mechanisms of disease. However, since they are rapidly cleared and do not persist in vertebrate hosts, it is intrinsically problematic to study the phenotype within *O. hermsi* ticks. Consequently, artificial feeding chambers would provide a useful method to obtain infected ticks. Although these mutants can be introduced to the tick by multiple methods, as discussed in the Introduction, none recapitulate the natural acquisition route of the pathogen by ingestion with a blood meal except artificial membrane chambers. Mammalian hosts develop relapsing fever by transmission from an infected arthropod. Therefore, an artificial feeding system for *O. hermsi* provides the ability to characterize relapsing fever strains at the critical points of acquisition and transmission by the tick. A widespread effort has generated artificial feeding systems for a variety of *Ornithodoros* species, but surprisingly not for *O. hermsi*.

A comparison of artificial feeding systems described for other *Ornithodoros* ticks identifies subtle but perhaps significant differences in protocols. For example, *O. coriaceus* were only induced to feed when mammal hair was placed on the surface of the membrane^5^, while the addition of rabbit ear wax to the membrane produced better results for *O. moubata* and *O. tholozani*^3^. In a separate study *O. moubata* ticks had a higher feeding efficiency with elevated temperatures (40° vs. 27 °C degrees)^10^. Other differences in feeding chambers included the membrane material (parafilm vs. silicone) and the addition of phagostimulants such as glucose, ATP, and glutathione^34,35^. These requirements may relate to differences in the biology of the *Ornithodoros* species being studied, or, alternatively, may reflect the peculiarities of lab-to-lab variation in technique. By adopting some of these phagostimulants and optimizing conditions we were able to induce later-stage nymphs and adult *O. hermsi* ticks to feed to engorgement in an artificial membrane chamber at a high efficiency (Table 1 and Fig. 1). The critical features for enticing the later stages of *O. hermsi* to feed appear to be the use of parafilm as the membrane material and incubation at 35 °C in a CO_2_ incubator (Fig. 2). Although adults fed well in a single experiment in a 37 °C water bath (Table 1), the results were inconsistent with nymphs, which only reached reproducibly high proportions of feeding under CO_2_ at 35 °C. The source of the blood (rabbit or bovine) did not seem to matter and adult ticks that fed on either produced viable eggs. Other kairomones and phagostimulants, such as the addition of mouse hair to the membrane and the inclusion of ATP and glucose in the blood, were not assessed individually for their necessity, but these conditions routinely yielded high repletion percentages (80–92%). The efficiency of tick feeding by this method is equivalent to that of ticks feeding on mice reported by McCoy et al. for nymphs and adults (79–92%)^38^.

One limitation to this method is the failure of larvae or 1st stage nymphs to feed. Possibly their mouthparts are too small to pierce the parafilm membrane, though the measured thickness of the stretched parafilm in our system is approximately 39 µm (standard deviation ± 9.4, N = 7 independent measurements). The reported length of *O. hermsi* larval hypostomes ranges from 62 to ~ 80 µm^42,43^ indicating that the mouthparts of these immature stages should be long enough to cross the parafilm. Alternatively, *O. hermsi* are relatively small ticks, even compared to other *Ornithodoros* species, and perhaps the tensile strength of the parafilm poses to great of a resistance to the forces that can be generated by the cheliceral digits of these small ticks. Of the *Ornithodoros* artificial feeding systems surveyed in the literature, only two described limited success with feeding larvae of *O. turicatae* and *O. puertoricensis*^4,7^. Neither paper noted significant changes to their protocol to stimulate larval feeding compared to that of the nymph or adult, but both had reduced success with larval feeding. Possibly the longer hypostomes of these species compared to that of *O. hermsi*^42,43^ permit them to generate the force necessary to penetrate the artificial membrane. We have been unable to definitively determine why the early stages of *O. hermsi* do not feed efficiently through artificial membranes.

The success with feeding of *O. hermsi* ticks encouraged us to assess pathogen acquisition using the feeding chambers. Of particular interest was whether we could use this system to infect ticks with mutant strains of *B. hermsii* attenuated for mammalian infection. Raffel et al. induced an artificial spirochetemia in a single mouse by injecting 1.5 × 10^8^ Vmp^–^ spirochetes and permitting ticks to feed before the mutant strain was cleared, demonstrating that Vmp was not necessary for either acquisition or persistence in the tick^25^. Using artificial membrane chambers we confirmed this finding by feeding *O. hermsi* on blood inoculated with either WT or Vmp^–^ *B. hermsii* strains. The densities of spirochetes detected in the blood meal ranged from 1 × 10^6^ to 6 × 10^7^ *B. hermsii*/mL, comparable to densities observed in spirochetemic mice^25,37,38^. We quantified spirochete numbers from the engorged ticks after feeding and demonstrated that both strains were acquired at equivalent densities by ticks (Fig. 3). McCoy et al. reported the blood volume ingested by *O. hermsi* 3rd stage nymphs and females averaged approximately 8 µL and 16 µL of blood, respectively^38^. Using these figures, we would predict that a female engorged on blood at a concentration of 1 × 10^7^ bacteria/mL would, on average, imbibe 1.6 × 10^5^ spirochetes (1 × 10^7^ bacteria/mL × 0.016 ml). The mean densities of *B. hermsii* WT and Vmp^–^ strains in ticks infected through artificial membrane feeding determined in this study were 1.2 × 10^5^ and 1.6 × 10^5^, respectively (Fig. 3), as predicted based on the measurements of blood imbibed by *B. hermsii*^38^.

Ticks infected with either *B. hermsii* strain successfully transmitted the infection to SCID mice and produced a spirochetemia (Fig. 4). Thus, ticks that had acquired *B. hermsii* through feeding on artificial membranes maintained the spirochetal infection through the molt and successfully transmitted the spirochetes to mice. When three infected ticks successfully fed on a mouse, all mice became infected. However, when a mouse was fed on by one or two ticks the mouse failed to become infected. These results are in agreement with those of Boyle et al., who demonstrated that three *O. turicata* ticks were sufficient to transmit *B. turicatae* to most mice in their study, whereas one or two ticks per mouse rarely transmitted the infection^44^. Davis and Walker reported slightly higher success with 65% of individual *O. hermsi* ticks transmitting *B. hermsii*^45^.

Alternatively, the lower number of infected SCID mice by the Vmp^–^ strain may result from the leaky B cell response that can occur in some SCID mice, which may impact the mutant strain more than the WT^46^. Regardless of the cause, our results indicate that three ticks per mouse were sufficient to transmit *B. hermsii* to all mice in this study. However, the spirochete densities in these ticks were substantially lower after the molt than at acquisition (Fig. 5 compared to Fig. 3). The reduction in spirochete numbers from the acquisition stage to the transmission stage was > 99%. As noted above, despite the decline in spirochete numbers, three ticks per mouse was still sufficient to ensure transmission.

The low densities of *B. hermsii* in ticks was a surprising result, although McCoy et al. noted an approximate 86% reduction of *B. hermsii* numbers in the five days following acquisition feeding^38^. In the Lyme disease spirochete that is vectored by hard ticks of the *Ixodes* genus, *B. burgdorferi* populations reach their highest densities shortly after the bloodmeal, but decline tenfold when assessed after the molt^47^. Our observation of spirochete densities decreasing over the lifespan of membrane-fed ticks dictates that future population dynamics studies be conducted comparing *B. hermsii* numbers within ticks that have fed upon mice versus those fed in membrane chambers.

In summary, the artificial membrane feeding chambers provide a new method for *O. hermsi* maintenance and insights into tick-microbe interactions. Adult ticks infected with WT or the Vmp^–^ mutant using this technique went on to produce viable egg clutches (Table 1), indicating that this artificial feeding system provides all necessary requirements for *O. hermsi* to develop through its life cycle. The observations of the *B. hermsii* Vmp^–^ mutant reinforces the evidence^25^ that Vmp is not required for survival in the tick but as an adaptation for mammalian infection. The artificial membrane feeding system for *O. hermsi* can be used for studies on pathogen transmission (e.g. number of microbes transmitted during feeding) and also for transstadial and transovarial transmission experiments. These chambers provide an efficient and inexpensive method for rearing *O. hermsi* ticks from second stage nymphs through adults that produce viable larvae, thereby significantly reducing the requirement for live animals. Additionally, this system affords a simple method to infect ticks with *B. hermsii* mutants that are incapable of persisting in mammals and opens new avenues for investigating the mechanism of vector specificity displayed by tick-borne relapsing fever spirochetes.

## Data Availability

All data generated or analyzed during this study are included in this published article.
